# Prevalence of rapid weight loss in Olympic style wrestlers

**DOI:** 10.1080/15502783.2022.2119095

**Published:** 2022-10-11

**Authors:** Roberto Roklicer, Carlo Rossi, Antonino Bianco, Valdemar Stajer, Marijana Ranisavljev, Nikola Todorovic, Marko Manojlovic, Barbara Gilic, Tatjana Trivic, Patrik Drid

**Affiliations:** a Faculty of Sport and Physical Education, University of Novi Sad, Novi Sad, Serbia; b Sport and Exercise Sciences Research Unit, University of Palermo, Palermo, Italy; c Faculty of Kinesiology, University of Split, Split, Croatia; d Faculty of Kinesiology, University of Zagreb, Zagreb, Croatia

**Keywords:** Combat sports, elite athletes, weight reduction methods

## Abstract

**Background:**

The methodology applied for rapid weight loss (RWL) among elite wrestlers is quite unexplored. Therefore, the aim of this study was to analyze the prevalence of sources of influence and methods used for RWL and to determine the differences between wrestling styles.

**Methods:**

A total of 229 wrestlers who competed at the World Championship held in Belgrade, Serbia, participated in this research. All respondents completed a questionnaire designed to evaluate RWL patterns in combat athletes. Participants were classified according to wrestling style: Greco-Roman, freestyle, and women wrestling.

**Results:**

Sixty-nine percent of wrestlers had previously lost weight to compete. Most respondents start losing weight approximately seven days before a competition. Athletes reported that they commonly reduced 3.84 ± 2.82 kg to reach the target weight. The wrestling coach represents the most influential person in terms of RWL strategies, while nutritionists and physicians have the least impact on the weight-cutting process. Regarding the methods applied, differences between all the three styles were found in the following variables: training in a heated room, restricting fluid ingestion, training in plastic suits, gradual dieting, increased exercise, diet pills, and sauna. The most frequently used techniques were increased exercise, gradual dieting, training in a heated room, and sauna for all competitors. Diet pills, diuretics, laxatives, and vomiting were the least implemented methods.

**Conclusions:**

The obtained results suggest that most wrestlers practice RWL despite the harmful health effects. The education of wrestling coaches is necessary in order to control and decrease the negative impact of RWL.

## 1. Introduction

Wrestling was an essential part of the Ancient Olympic Games [1], so it is considered one of the oldest combat sports (CS) on the planet. It is a sport consisting of high-intensity efforts interspersed with short bouts of mild-to-moderate-intensity work [2]. The rules have been changing throughout history. However, despite all those changes, the power techniques that require strength and explosiveness have always been demanded from the athletes [3]. Consequently, modern wrestling relies a lot on the anaerobic energy system [4], which tends to dramatically increase heart rate to near-maximal levels [5]. It tends to accumulate significant lactate concentrations (10–20 mM) [6].

There are three styles of wrestling included in the Olympic Games program: Greco-Roman, freestyle, and wrestling for women. Previous research has shown no major physiological differences between Greco-Roman and freestyle [2]. Each wrestling style is characterized by intermittent, short-duration, and high-intensity actions lasting a total of 6 min (two rounds of 3 min with the 30 s break in between). Anaerobic power is considered fundamental by frequently employing explosive techniques, thus making the wrestlers capable of winning the match before the end of the second round [7].

The rules of freestyle wrestling, for both men and women, are the same in terms of sports regulations. In this style, athletes are allowed to use their legs to perform technical activities and actions on the opponent’s legs [8]. Thus, the ability to use techniques to gain advantage and win are identical for both genders. However, the rules of Greco-Roman-style wrestling are different, as athletes are allowed to use only upper body techniques, excluding techniques of grabbing legs and tripping.

Furthermore, the tactical preparation for the match against top-level athletes can be similar for both men and women [8]. Athletes in both Greco-Roman and freestyle wrestling are divided into 10 official weight categories, of which six are included in the Olympics. Due to the differences in body weight between the genders, different weight categories have been introduced for male and female wrestlers [8].

Most of the wrestlers tend to increase the total lean tissue mass and reduce the amount of fat body mass, thus minimizing the total body weight prior to competition [9]. In addition to losing body fat, reducing total body water also leads to a reduction in total body weight. Consequently, athletes tend to achieve their target weight with an aim to compete in the lowest weight class possible. By doing so, it is presumed that combat sport (CS) athletes might gain both a psychological and competitive advantage over their lighter opponents [10,11]. Rapid weight loss (RWL) is a procedure characterized by a temporary weight loss of at least 5% of body weight a few days ahead of the official weigh-ins [12]. Regardless of the type of combat sport practiced, there are different methods of inducing RWL. Due to internal or external factors, methods such as prolonged fasting, skipping meals, or reduced fluid intake are usually applied [13]. Additionally, procedures such as high-intensity training sessions, training with plastic suits, and use of sauna are frequently used [13–16].

A recent study found that the RWL presents one of the most serious problems in combat sports, and one of the findings that impressed authors the most was the early age at which grapplers started practicing weight loss [17]. Another study, carried out by Figlioli et al. [10], showed that most sambo athletes, both junior and senior, adopt RWL practices before competition by skipping meals, using a gradual diet, sauna sessions, and training in plastic suits. Thus far, the literature has reported mostly detrimental health implications of RWL on CS athletes’ health, such as kidney function impairment, total mood disturbance, and damaging muscle tissue [18–21]. The fact that employment of RWL goes beyond the borders of extreme was proven by the case of a 5-year-old boy who went through such a procedure in order to compete in a certain weight category [22]. Additionally, there are unfortunate cases of RWL procedures undergone by three collegiate wrestlers who lost a substantial amount of weight in the weeks before the competition. Regrettably, due to the consequences of overheating, they could not avoid lethal outcomes [23]. Having in mind all this evidence, the question of banning RWL from combat sports was raised [12]. Nevertheless, aggressive methods are still used today to potentially bring the wrestler to a competitive advantage.

The goal of this study was to identify the prevalence of RWL techniques and compare the various methods practiced by Greco-Roman, freestyle, and women wrestlers.

## 2. Methods

### 2.1. Experimental design

To assess RWL methods among wrestlers, a validated RWL questionnaire created by Artioli et al. [24] was applied. This self-reporting questionnaire is designed to evaluate RWL patterns consisting of 21 items related to personal information, competitive level, nutrition status, RWL history, and RWL behaviors. To facilitate data collection, the questionnaires were translated from the original Portuguese language to several languages (e.g. Russian, Italian, Spanish, French, Serbian, Romanian, German, and Bulgarian). The questionnaires were completed anonymously. Furthermore, examiners were ready to provide more detailed information related to the questionnaires and answer all additional questions throughout the procedure if necessary.

### 2.2. Participants

The study sample consisted of 229 top-level Greco-Roman and Freestyle, both men and women wrestlers, who participated in the U-23 World Championships held in Belgrade 2021. A total of 49 countries took part in the competition. Participants were divided in accordance with the wrestling style they were competing in, as follows: Greco-Roman (*n* = 72, mean weight 81.41 ± 20.05 kg; mean height 175.52 ± 10.25 cm; mean age 20.94 ± 1.66 years), freestyle (*n* = 62, mean weight 78.18 ± 17.61 kg; mean height 172.56 ± 17.27 cm; mean age 21.32 ± 1.50 years), and women wrestling (*n* = 95, mean weight 64.89 ± 10.14 kg; mean height 167.01 ± 9.44 cm; mean age 20.67 ± 1.69 years). This study was conducted in accordance with the Declaration of Helsinki [25], and ethical approval from the ethical board of the University of Novi Sad, Serbia (Ref. No. 46-06-02/2020-1) was obtained. The examiners have thoroughly explained the objectives of our study to the participants. All the respondents participated voluntarily in this study by signing the written informed consent.

### 2.3. Statistical analysis

All the data were analyzed with SPSS (Statistical Package for Social Sciences ver. 24.0, IBM Statistics, Armonk, NY, USA). The normality of the data was checked using a Kolmogorov–Smirnov test. Calculation of the descriptive statistics included all the variables elaborated in the analysis, including weight, height, RWL frequency, RWL methodology, and the influence in weight-cutting practice. Additionally, to detect the differences between various wrestling styles, the Kruskal–Wallis test with Bonferroni correction for multiple comparisons was applied. The variety of RWL techniques applied and source of influence between the groups were calculated by using the Chi-Square test. The level of significance was set at *p* ≤ 0.05.

## 3. Results

From the total sample included in the study, 69% reported making weight prior to the competition. Wrestlers usually start losing weight seven days before the official weigh-in. Respondents reported that they commonly reduce 3.84 (±2.82) kg for achieving the target weight. Competitors stated that they started conducting rapid weight loss procedures around 15 years of age (Table 1).

**Table 1.** Rapid weight loss (RWL) history.

	Group	Mean ± SD	Mean rank	*p* Value
At what age did you begin to compete wrestling?	Greco-Roman	11.42 ± 2.78	99.17	0.000***
	Women wrestling	13.03 ± 3.01^###§^	136.51	
	Freestyle	11.07 ± 2.96	93.86	
How many days before the competition do you usually cut weight? (days)	Greco-Roman	9.80 ± 8.53	98.70	0.099
	Women wrestling	7.67 ± 7.96	82.40	
	Freestyle	6.69 ± 6.13	80.24	
How much weight do you usually cut before the competition? (kg)	Greco-Roman	3.84 ± 2.89	88.75	0.012*
	Women wrestling	3.53 ± 3.36^#^	72.96	
	Freestyle	4.17 ± 2.23	99.98	
At what age did you start to cut weight before the competition? (years)	Greco-Roman	14.7 ± 3.74	85.52	0.076
	Women wrestling	15.18 ± 4.20	97.31	
	Freestyle	14.07 ± 3.63	75.65	
The greatest amount of weight lost for the competition?	Greco-Roman	6.72 ± 2.77	99.59	0.000***
	Women wrestling	4.21 ± 2.80^###§^	57.99	
	Freestyle	6.37 ± 2.85	93.66	
How much weight do you usually regain after the competition? (kg)	Greco-Roman	4.59 ± 4.12	103.80	0.000***
	Women wrestling	2.86 ± 2.96^##§^	66.60	
	Freestyle	3.38 ± 1.40	97.31	

*** statistically significant difference, *p* ≤ 0.001; * statistically significant difference, *p* ≤ 0.05; #significantly different compared to freestyle wrestlers, *p* ≤ 0.05; ## significantly different compared to freestyle wrestlers, *p* ≤ 0.01; § significantly different compared to Greco-Roman wrestlers, *p* ≤ 0.001.

**Figure 1.**
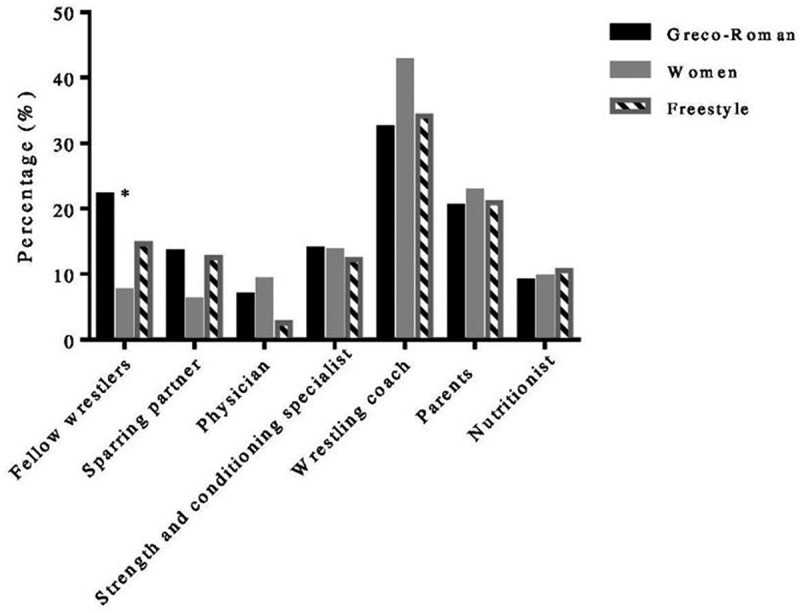
Source of influence of RWL on wrestlers. * Significant difference between the groups, *p* ≤ 0.05.

The most influential person in RWL strategies turned out to be the wrestling coach for all three examined styles (Greco-Roman, 32.3%; Freestyle, 34.1%; and Women, 42.6%). The least influence on weight-cutting process had nutritionist for the Greco-Roman and women (55.4% and 65.1%, respectively) and physician for freestyle wrestlers (50%). This was determined by the participants’ answers, “very influential” and “not influential”(Figure 1). In terms of the influence, there were no statistically significant differences among different styles of wrestlers except for the variable “fellow wrestler.”

**Figure 2.**
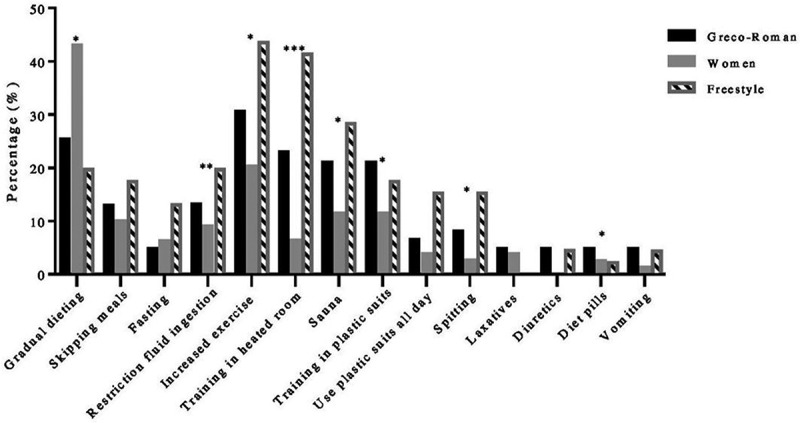
Methods used by athletes during RWL. * Significant difference between the groups, *p* ≤ 0.05; ** significant difference between the groups, *p* ≤ 0.01; *** significant difference between the groups, *p* ≤ 0.001.

In terms of RWL methods used, significant differences between the three styles were observed in the following variables: training in heated room (*p* = 0.000), restricting fluid ingestion (*p* = 0.004), *s*pitting (*p* = 0.014), training in plastic suits (*p* = 0.015), gradual dieting (*p* = 0.017), increased exercise (*p* = 0.027), diet pills (*p* = 0.049), and sauna (*p* = 0.050) (Figure 2).

For Greco-Roman wrestlers, the most frequently (answer “always”) used methods were increased exercise (30.6%), gradual dieting (25.4%), and training in a heated room (23%), while the least used methods (answer “never”) were using diet pills and vomiting (75.8%) and laxatives (74.2%). Freestyle wrestlers reported increased exercise (43.5%), training in a heated room (41.3%), and using sauna (28.3%) as the most frequent methods. The least frequently used methods reported by freestyle wrestlers were vomiting (69.6%), using diet pills (67.4%), and diuretics (62.6%). Women most often used gradual dieting (43%), increased exercise (20.3%), training in plastic suits, and sauna (11.4%) in order to achieve the desired weight class. The methods used least by female competitors were reported to be diuretics (74.7%), laxatives (69.6%), and diet pills (64.6%).

## 4. Discussion

The goal of this study was to determine the prevalence of RWL methods and delineate the differences between Greco-Roman women and freestyle wrestling. The results of our study suggest that the majority (69%) of the athletes usually adopt weight loss techniques prior to competition. However, there were some differences between the athletes belonging to different wrestling styles in terms of RWL history data, source of influence, and methods.

Greco-Roman and freestyle wrestlers started to compete at approximately similar ages, while women started somewhat later. Thus, the age-related difference was observed due to athletes’ gender. This was also evident for other items associated with both average and the greatest amount of weight loss before the competition and the amount of weight regained within the week following the competition, as higher values were obtained for male competitors (both Greco-Roman and freestyle). A significant gender difference was observed in gradual dieting as women reported a higher percentage of this method compared to Greco-Roman and freestyle wrestlers, who usually conduct more aggressive weight loss procedures. Females in this study also reported practicing methods such as spitting, using sauna, plastic suits, and training in heated rooms, as well as increased exercise considerably less than male athletes. Additionally, females also barely restrict fluid injection compared to male counterparts.

A study conducted by Artioli et al. [12] revealed lower average weight reduced by judokas compared to wrestlers of our study (1.6 ± 1.6 vs. 4.1 ± 2.2 kg). Additionally, the most weight loss was also greater in our sample than in judo athletes (6.3 ± 2.8 vs. 4.0 ± 3.1). Judokas appeared to start losing weight slightly earlier in comparison to wrestlers (12.6 ± 6.1 vs. 14.07 ± 3.63 years). Weight regained in the first week after the competition was higher in wrestlers than in judo athletes (3.3 ± 1.4 vs. 1.6 ± 1.4 kg). However, the number of days in which the weight is usually lost was in line with our study (approximately seven days). These differences could be explained due to the age difference between the two samples and their competitive level.

A study done by Drid et al. [26] investigated the RWL methods used in elite sambo athletes. The authors found that 87% of the total sample have reported cutting weight intentionally prior to competition, with an average moderately higher weight (5.27 ± 7.57 kg) than wrestlers included in our study. The most prevalent methods were gradual dieting, sauna use, and skipping meals, which can be comparable to female wrestlers from our study. The authors of this study stated that a greater amount of weight regained by male athletes was observed, which is analogous to the results of our study.

The proof that combat sports athletes undergo the RWL methods also at a younger age can be found in a study carried out by Berkovich et al. [27]. The percentage of male adolescent judokas reporting RWL in that study was quite high as 80%. The most prevalent method among the participants was increased exercise, followed by gradual dieting and skipping meals. Having this in mind, it could be observed that the younger athletes use exercise and diet rather than a heated environment to cut their weight. Similar results were observed among a large sample of Iranian wrestlers as they reported increased exercise and eating less food as predominant RWL methods [28].

The RWL is practiced in striking combat sports as well. Namely, da Silva Santos et al. [29] examined the prevalence of weight-loss strategies among taekwondo athletes. When the sample of international-level taekwondo competitors were compared to our study participants, wrestlers, on average, started to compete earlier and lose more weight before the competition. Additionally, our respondents have reported a greater amount of most weight loss prior to competition and greater weight regains in a week after. The most prevalent method reported by taekwondo athletes was increased exercise. These observations were evident for both men and women. Another study exploring elite kickboxers [30] indicated that the majority of athletes used gradual dieting as a dominant method, which is in line with female subjects from our study. The most influence on weight management behavior had a coach and fellow athlete, which is also consistent with our study results. The reason for this could be that the competitor spends most of his time with either a coach or his teammate while preparing for the competition. A recent study on grapplers found that female grapplers use less sauna and heated room to cut weight than their male counterparts [17]. These results are in accordance with our study as well. On the other hand, grapplers seem to start using RWL procedures considerably later compared to wrestlers (19 vs. 15 years, respectively) [17].

Gradual dieting was the most prevalent method among both men and women, which is in line with female wrestlers’ reports. Another research done by Figlioli et al. [10] revealed gradual dieting, increased exercise, and training in a heated environment as leading RWL methods for senior sambo athletes, while the coach and teammate were the most influential persons. However, the difference in the influence of fellow wrestlers among males and females differs in our study since 47.1% of female participants answered for the teammate as non-influential. In our study, strength and conditioning specialists seem to have much less influence than one could assume. In such type of sport, it is usually the head coach (wrestling coach) who takes care of both conditioning level and technical-tactical preparation of the athlete. On the other hand, the high percentage of parents’ influence can be a consequence of their support, while athletes spend time with them, particularly the last few days prior to competition. Regrettably, the low impact of physicians and nutritionists is present among all the three investigated styles, which is in line with other authors’ results [12,17]. Considering the myriad of deleterious effects of RWL, participation of these professions should be more involved in RWL programs.

Although the detrimental effects of acute weight reduction in combat sports athletes are widely known, most studies have reported that a high percentage of athletes still undergo these methods. Speaking of RWL strategies, the emphasis should be on maintaining the health of the athletes and preventing harmful effects as much as possible. Having this in mind, combat sports athletes should consider implementing more suitable and safer RWL techniques such as “water loading” prior to fluid restriction since this method does not appear to have adverse implications on blood chemistry changes and physical performance [31].

This research has several limitations. The main limitation of this study comes from its cross-sectional design; hence, the causality cannot be determined. Further, the informative questionnaire is the main measuring tool in this study, as we did not include an assessment of body composition and biochematological markers. Also, the limitation comes from using the questionnaire as participants could be susceptible to providing socially desirable answers and avoid the true responses, especially for questions regarding using prohibited and dangerous methods. However, we tried to reduce and eliminate this problem by making the questionnaire anonymous. Even though the questionnaire was translated to the languages of the respondents, they reported that some questions were not completely understood. Thus, those questions should be identified and further adjusted to overcome the language barriers in future studies.

This study has several strengths. Namely, this is probably the first study conducted on a representative sample of wrestlers (U23), including all three Olympic-style athletes. Indeed, we included athletes of the elite competitive level, all international competitors.

The present study results show the current state of weight loss commonness among elite wrestlers of every Olympic style. These results depict the most practiced methods, which may likely have an adverse impact on athletes’ health. Accordingly, the education of coaches is of critical importance. Such educational programs could provide useful information on both physiological and other types of athletes’ responses that could possibly affect their performance and health. In this manner, coaches around the world could likely become able to give better and safer advice to their competitors when it comes to acute reduction of weight. In the future, sporting organizations and institutions, along with sports scientists, should strive to modify the “weight cutting” rules to a certain extent in order to prevent all possible harmful effects on health.

## 5. Conclusions

To our knowledge, this is the first study conducted on a representative sample of wrestlers (U23), including all three Olympic-style athletes.

The results obtained suggest that most of the wrestlers still undergo the RWL despite the well-known detrimental effects. Nevertheless, slightly different methods were observed between male and female athletes, while men competing in both styles practically applied the same weight loss techniques. Given that the coaches have the most influence on wrestlers, there should be education provided for them to modify the methods and make them the safest possible.
